# Correction

**DOI:** 10.1080/19932820.2025.2475687

**Published:** 2025-03-09

**Article title**: miR-92a promotes proliferation and inhibits apoptosis of prostate cancer cells through the PTEN/Akt signaling pathway

**Authors**: Zheng Yanshen, Yang Lifen, Wu Xilian, Dong Zhong and Mai Huihong

**Journal**: *Libyan Journal of Medicine*

**Bibliometrics**: Volume 16, Number 01, pages 1-8

**DOI**:https://doi.org/10.1080/19932820.2021.1971837

The authors have noted that some of the figures were duplicated after publication. After data verification and screening, the result images have been replaced accordingly. The corrected images for Figures 2, 3, and 4 are provided below. This correction does not affect the description, interpretation, or original conclusions of the article. The authors apologize for any inconvenience caused. I would like to request a correction regarding some of the figures in my paper titled “miR-92a promotes proliferation and inhibits apoptosis of prostate cancer cells”, published in your journal. Upon reviewing the final manuscript, I found that certain images are duplicated.

**Figure 2:**
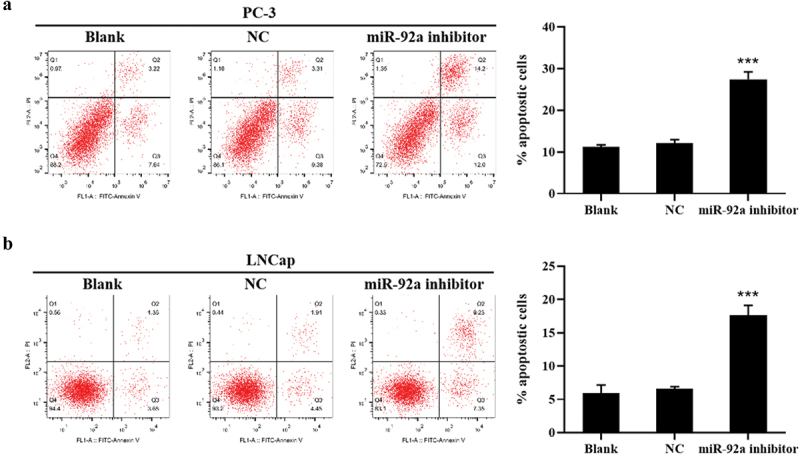

**Figure 3:**
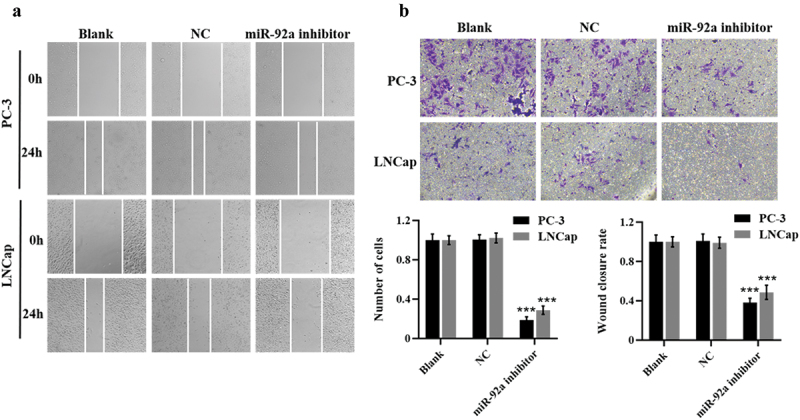

**Figure 4:**
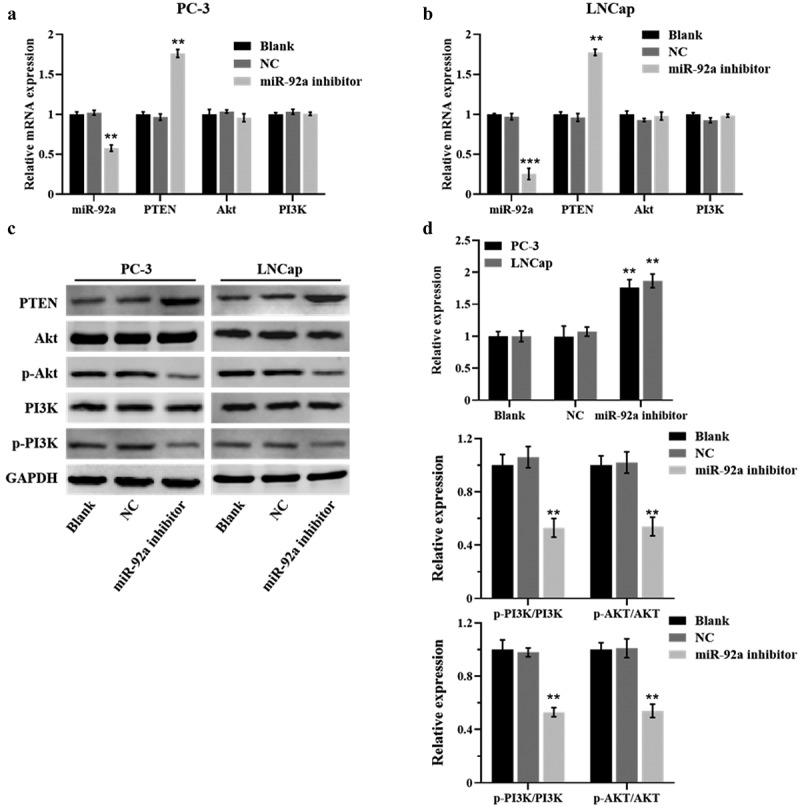

After data verification and screening, the result images have been replaced accordingly.

**Correct**

